# Blood spinal cord barrier disruption recovers in patients with degenerative cervical myelopathy after surgical decompression: a prospective cohort study

**DOI:** 10.1038/s41598-023-34004-2

**Published:** 2023-05-06

**Authors:** Tobias Philip Schmidt, Kerstin Jütten, Ulf Bertram, Lars Ove Brandenburg, Thomas Pufe, Daniel Delev, Alexander Gombert, Christian Andreas Mueller, Hans Clusmann, Christian Blume

**Affiliations:** 1 Department of Neurosurgery, RWTH Aachen University, Pauwelsstrasse 30, 52074 Aachen, Germany; 2 Institute of Anatomy and Cell Biology, RWTH Aachen University, Wendlingweg 2, 52074 Aachen, Germany; 3 Department of Vascular Surgery, RWTH Aachen University, Pauwelsstrasse 30, 52074 Aachen, Germany

**Keywords:** Neurodegeneration, Regeneration and repair in the nervous system, Molecular neuroscience

## Abstract

The pathophysiology of degenerative cervical myelopathy (DCM) is characterized by chronic compression-induced damage to the spinal cord leading to secondary harm such as disruption of the blood spinal cord barrier (BSCB). It is therefore the purpose of this study to analyze BSCB disruption in pre- and postoperative DCM patients and to correlate those with the clinical status and postoperative outcome. This prospectively controlled cohort included 50 DCM patients (21 female; 29 male; mean age: 62.9 ± 11.2 years). As neurological healthy controls, 52 (17 female; 35 male; mean age 61.8 ± 17.3 years) patients with thoracic abdominal aortic aneurysm (TAAA) and indication for open surgery were included. All patients underwent a neurological examination and DCM-associated scores (Neck Disability Index, modified Japanese Orthopaedic Association Score) were assessed. To evaluate the BSCB status, blood and cerebrospinal fluid (CSF) samples (lumbar puncture or CSF drainage) were taken preoperatively and in 15 DCM patients postoperatively (4 female; 11 male; mean age: 64.7 ± 11.1 years). Regarding BSCB disruption, CSF and blood serum were examined for albumin, immunoglobulin (Ig) G, IgA and IgM. Quotients for CSF/serum were standardized and calculated according to Reiber diagnostic criteria. Significantly increased preoperative CSF/serum quotients were found in DCM patients as compared to control patients: Albumin_Q_ (*p* < .001), IgA_Q_ (*p* < .001) and IgG_Q_ (*p* < .001). IgM_Q_ showed no significant difference (*T* = − 1.15, *p* = .255). After surgical decompression, neurological symptoms improved in DCM patients, as shown by a significantly higher postoperative mJOA compared to the preoperative score (*p* = .001). This neurological improvement was accompanied by a significant change in postoperative CSF/serum quotients for Albumin (*p* = .005) and IgG (*p* = .004) with a trend of a weak correlation between CSF markers and neurological recovery. This study further substantiates the previous findings, that a BSCB disruption in DCM patients is evident. Interestingly, surgical decompression appears to be accompanied by neurological improvement and a reduction of CSF/serum quotients, implying a BSCB recovery. We found a weak association between BSCB recovery and neurological improvement. A BSCB disruption might be a key pathomechanism in DCM patients, which could be relevant to treatment and clinical recovery.

## Introduction

Degenerative cervical myelopathy (DCM) is a result of a chronic mechanical harm to the spinal cord leading to a composition of highly complex pathomechanisms on the molecular and cellular level^1,2^. As part of the central nervous system, the spinal cord has a special barrier along vessels, the blood-spinal cord barrier (BSCB)^3^. This BSCB protects the spinal cord mechanically and biochemically from substances and cells within the intravascular compartment, thereby maintaining a healthy microenvironment^4^. In a physiological state, proteins such as albumin and immunoglobulin (Ig) can only pass from the blood to the spinal cord tissue by passive diffusion in capillaries. Larger vessels prevent this passage through the BSCB, resulting in a characteristic CSF/serum quotient for each protein^5^. It is known that a structural damage of the BSCB is associated with increased permeability leading to protein efflux and edema (among other effects). The result is a self-sustaining cascade of secondary injury to the spinal cord, as described in acute spinal cord injury^3,4,6^. The pathomechanism of BSCB disruption is responsible for an extended damage of the spinal cord beyond the local mechanical injury, including inflammatory cascades, increased macrophage activation, Wallerian degeneration and cell death^7–11^. Already investigated extensively in traumatic spinal cord injury (SCI), BSCB disruption is still underestimated in chronic degenerative circumstances^12^. This key pathomechanism seems to be an important component in DCM, also providing possible future treatment options.

BSCB disruption has been in the focus of our previous publication, demonstrating its presence in DCM patients, giving first evidence of BSCB disruption in preoperative DCM patients with a distinct association with the clinical status of the patients^12^. To gain a deeper understanding of these promising results, the current study aims to replicate previous findings indicating a BSCB disruption in DCM patients, and additionally addresses the postoperative status.

## Methods

### Study procedure and sample analysis

The study was approved by the local ethics committee of the Medical Faculty of the RWTH Aachen University (EK 164/13) including the following amendment. Before the investigation, all participants gave written informed consent according to the Declaration of Helsinki (Medical Association 2008). Any participants who had a neurological condition other than DCM (e.g., neurodegenerative diseases, ischemic diseases, cerebral hemorrhage, central nervous system infections or spinal trauma) were excluded from participation in the study. All included patients underwent a neurological examination and the objective functional status was assessed by an experienced spine surgeon using the modified Japanese Orthopedic Association score (mJOA; normal function: 18 points, mild myelopathy: 15–17 points, moderate myelopathy: 12–14 points, severe myelopathy: 0–11 points) and the Neck Disability Index (NDI)^13–15^.

As described in a previous publication from 2020, our treatment recommendations were based on the AOSPINE guidelines for DCM^12,16^. We offered decompressive surgery as first-line therapy to DCM patients with moderate (mJOA 12–14) and severe (mJOA 0–11) clinical signs of myelopathy who also had correlating degenerative cervical spinal stenosis on imaging. In patients with mild signs of myelopathy (mJOA 15–17), surgery or conservative treatment with structured rehabilitation were recommended as possible option. In case of clinical deterioration, surgical intervention was strongly recommended. In DCM patients, cerebrospinal fluid (CSF) samples were obtained preoperatively by lumbar puncture (LP) or during CT myelography (if magnetic resonance imaging was contraindicated, e.g. with cardiac pacemaker). Three months after surgery (mean 121 ± 27 days), patients were electively examined in our outpatient clinic and a second LP was performed. According to our prior study, we included neurological healthy control patients^12^. These patients had an indication for open surgery concerning a thoracic abdominal aortic aneurysm (TAAA). Therefore, control patients routinely received a preoperative CSF drainage placement for intra- and postoperative intrathecal pressure monitoring^17^.

Blood serum samples of each patient were collected simultaneously with the corresponding CSF sample in all patients to perform Reiber diagnostics for detection of a BSCB disruption. A Queckenstedt maneuver was carried out in each DCM patient to rule out completely abolished CSF passage^18^. In addition, all CSF samples were examined for cytoalbuminous dissociation^19^.

The simultaneously collected CSF and blood serum samples were taken directly to the laboratory for examination. Routine laboratory values of CSF were determined: (1) CSF cell count (/µl), (2) lactate (mmol/l), and (3) protein concentration (g/l). CSF and blood serum samples were additionally analyzed for albumin, IgG, IgA and IgM (all mg/dl) by simultaneous nephelometric quantification (BN ProSpec System, Siemens Healthineers). Quotients (Q) of CSF/serum were calculated according to the standardized Reiber diagnostic criteria for Albumin_Q_, immunoglobulin G (IgG)_Q_, IgA_Q_ and IgM_Q_ (all Q: n × 10^–3^)^20^. Individual age-related references of Albumin_Q_ were calculated using the formula: (4 + age/15) × 10^–3^^21,22^. There were missing values for the following variables relevant to the preoperative/postoperative comparison: 1 for AlbuminQ, 1 for IgG, 1 for IgA and 7 for IgM.

**Figure 1:**
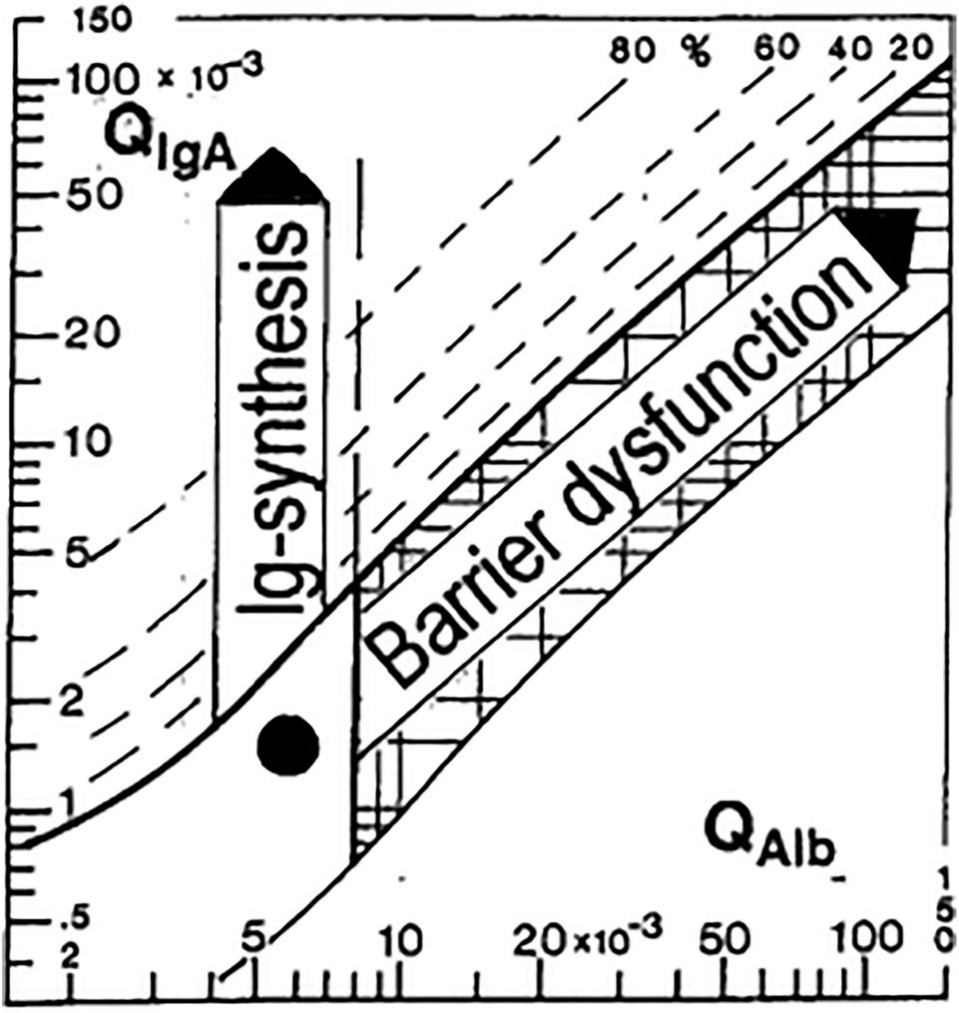
Reiber diagram. Please note that the black circle in the lower left area represents a normative ratio between Albumin_Q_ (QAlb in the figure) and IgA_Q_ (QIgA in the figure). A barrier dysfunction and Ig synthesis are marked in the graph published by Reiber et al.^5^.

The differentiation of a barrier disturbance from intrathecal synthesis is determined by the ratio of IgG_Q_ and Albumin_Q_, which is shown graphically in a Reiber diagram (Fig. 1). Values within the IgG_Q_ and outside the Albumin_Q_ reference range indicate a barrier disorder. Conversely, intrathecal synthesis is present if the values are outside the IgG_Q_ and inside the Albumin_Q_ reference range.

### Data analyses

All statistical analyses were performed with SPSS 25 (IBM Corp. Released 2016. IBM SPSS Statistics for Windows, Version 24.0. Armonk, NY: IBM Corp.). Data measures deviating more than 1.5 standard deviations (SD) from the group-specific mean were regarded as outliers and corrected for by being replaced by the “worst” group-specific score on that respective variable. Explorative analyses revealed that this was the case for pre-operative IgM_Q_ in two TAAA patients as well as for pre-operative IgA_Q_, IgG_Q_, IgM_Q_, and Albumin_Q_ in three DCM patients. In addition, another TAAA patient revealed extreme deviating values with regard to all parameters of interest (mJOA, IgA_Q_, IgG_Q_, IgM_Q_, and Albumin_Q_), so this patient was excluded from further analyses. All statistical comparisons were tested two-sided with a significance level of *p* < 0.05 and Bonferroni-corrected for multiple testing.

Differences in mJOA as well as CSF/serum coefficients between patients and controls were explored by applying Independent Samples t-Tests, including group (DCM, TAAA) as between-subject factor, and the variable of interest (mJOA, IgA_Q_, IgG_Q_, IgM_Q_, and Albumin_Q_) as dependent variable (adjusted *p* < 0.05 / 5 = 01).

Subgroup differences in CSF/serum coefficients between DCM patients with severe and non-severe mJOA scores were analyzed by means of Independent Samples t-Tests, including group (mJOA severe, mJOA non-severe) as between-subject factor, and the coefficient of interest (mJOA, IgA_Q_, IgG_Q_, IgM_Q_, and Albumin_Q_) as dependent variable (adjusted *p* < 0.05 / 5 = 01).

Patients’ intra-individual changes from pre- to postoperative assessment were analyzed using Paired Samples t-Tests, including the pair of interest (pre-post for each mJOA, NDI, IgA_Q_, IgG_Q_, IgM_Q_, and Albumin_Q_) as dependent variable (adjusted *p* < 0.05 / 6 = 008).

In order to assure that age as a possible confounding variable would not obscure analyses results, bivariate correlation analyses were performed, including age, mJOA, NDI, pre-/post-operative IgAQ, IgGQ, IgMQ, and AlbuminQ, tested two-sided and Bonferroni-corrected for multiple testing (adjusted *p* = 0.005). Results revealed no significant association between age and any variable of interest (all *p* > 0.05).

## Results

### Description of the study groups

Fifty DCM patients and 52 TAAA patients as neurologically healthy control group were included in this study. Due to rejected or unsuccessful LP, as well as unsuccessful lumbar drainage, 44 DCM and 46 TAAA patients remained for analysis. Baseline group characteristics (age, gender and comorbidities) and preoperative neurological scores (mJOA and NDI score) are shown in Table 1. As expected, there were significant group differences in neurological characteristics. There were 18 DCM patients with a mild paresis and 7 with a severe paresis (0–2/5 degree of strength) compared to the neurological healthy control group of TAAA patients. 25 DCM patients showed no paresis. A similar picture was seen with regard to ataxias: 38 patients with and 10 patients without ataxia in the DCM group compared to neurological healthy control group of TAAA patients. After approval of the amendment, a total of 15 patients could be included to receive a CSF puncture about three months after surgery.

**Table 1:** Baseline characteristics showing demographic, clinical findings and major comorbidities. TAAA = thoracic abdominal aortic aneurysm. DCM = degenerative cervical myelopathy. Pre = preoperative. SD = standard deviation. mJOA = modified Japanese Orthopedic Association. NDI = Neck Disability Index.

	TAAA		DCM		TAAA vs. DCM pre
	Male	Female	Male	Female	p value
Gender	35	17	29	21	0.331

	Mean	Sd	Mean	Sd	p value
Age	61.1	16.7	62.9	11.2	0.529

	n	%	n	%	
Diabetes	4	7.7	8	16.0	0.358
High blood pressure	38	73.1	27	54.0	0.004
Nicotine addiction	18	34.6	24	48.0	0.410
mJOA pre	17.7	0.6	10.7	2.8	< 0.001
NDI pre	6.1	8.3	40.4	20.8	< 0.001

### (1) Neurological status

#### Comparison of preoperative clinical conditions in DCM patients and TAAA

**Figure 2:**
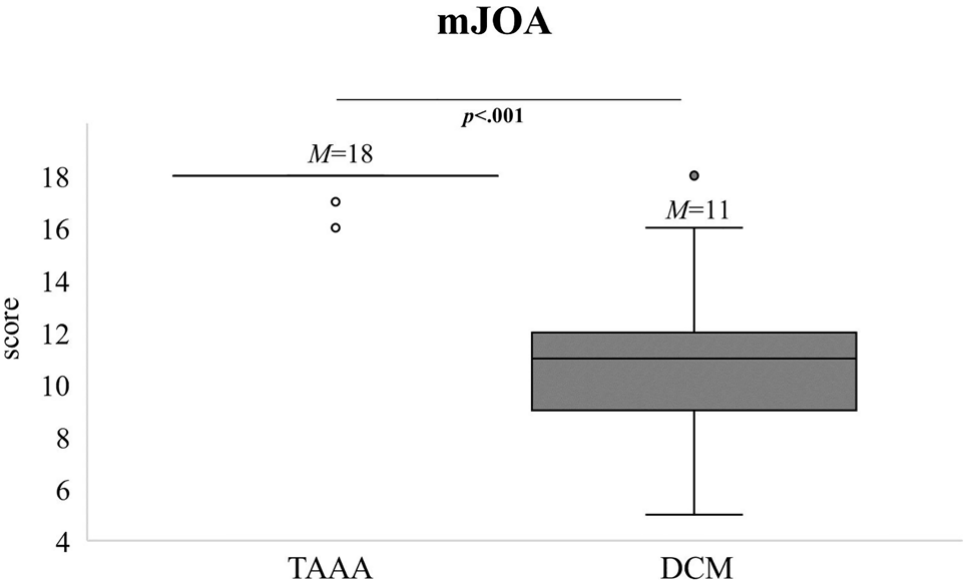
Extent of clinical disturbance as myelopathy preoperatively. As expected, significantly lower mJOA score were detected in the DCM group (n = 49) compared to the TAAA group (n = 48). M = mean. p = p value. mJOA = modified Japanese Orthopedic Association. TAAA = thoracic abdominal aortic aneurysm. DCM = degenerative cervical myelopathy.

As expected, a significant difference between the two groups was evident for the clinical NDI score (*p* < 0.001, data not shown) and the mJOA score (*p* < 0.001, Fig. 2). In total, seven patients were classified as mildly affected (mJOA 15–17), 13 as moderately (mJOA 12–14) and 30 as severe affected (mJOA 0–11).

#### Comparison of clinical conditions in DCM patients with preoperative and postoperative assessment and TAAA patients

**Figure 3:**
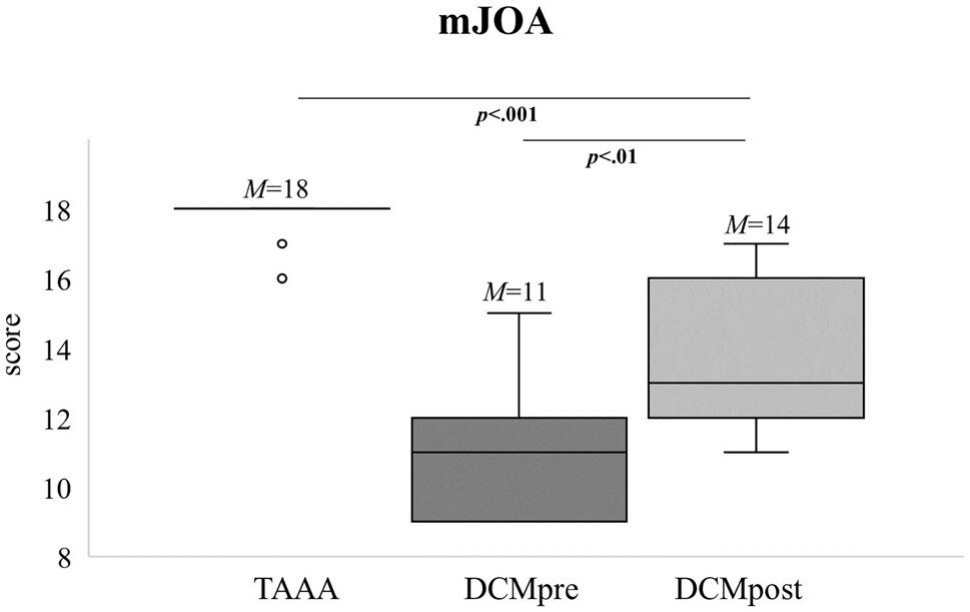
Extent of clinical myelopathy pre- and postoperatively. As expected, we detected a significant difference between pre- and postoperative DCM patients (n_pre/post_ = 16). While there are still significant differences between control patients (n = 48) and postoperative DCM patients, please note the trend of convergence. M = mean. p = p value. mJOA = modified Japanese Orthopedic Association. TAAA = thoracic abdominal aortic aneurysm. DCM = degenerative cervical myelopathy. Pre = preoperative. Post = postoperative.

As shown in Fig. 3, we detected a significantly higher mJOA score postoperatively compared with the preoperative score (*p* = 0.001). However, the difference between DCM patients’ postoperative mJOA score and the mJOA score of the TAAA group remained significant (*p* < 0.001), albeit with a tendency to approach the values of the neurologically healthy patients. NDI score was significantly lower postoperatively as compared to preoperative assessment (*M*pre = 40, *M*post = 30; *p* = 0.007, data not shown).

### (2) CSF findings

#### Comparison of preoperative CSF/serum quotients in DCM patients and TAAA patients

**Figure 4:**
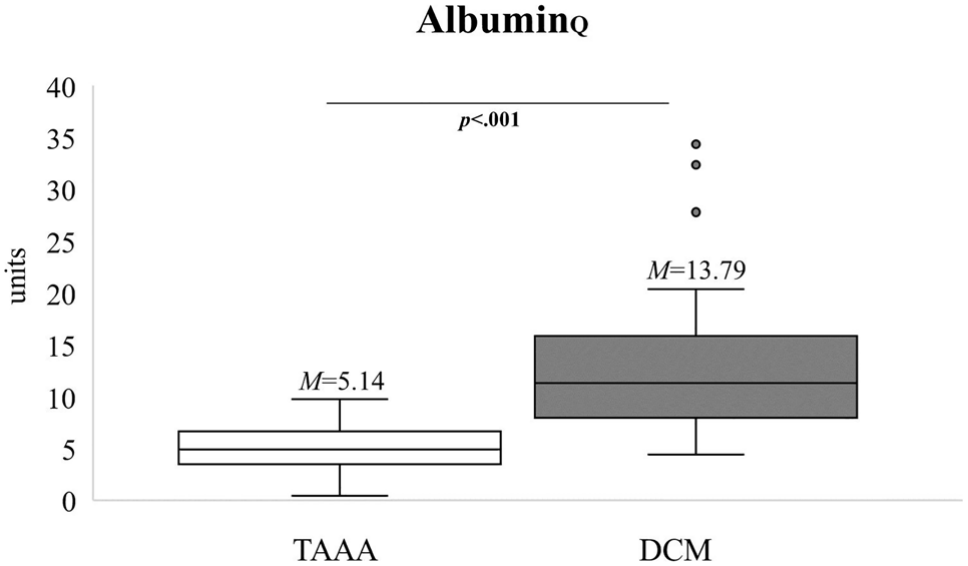
Extent of blood spinal cord barrier (BSCB) disruption preoperatively. The quotient for albumin (Albumin_Q_) is shown and compared between the control (n = 43) and DCM groups (n = 43). Please note the significant difference indicating BSCB disruption. M = mean. p = p value. TAAA = thoracic abdominal aortic aneurysm. DCM = degenerative cervical myelopathy.

**Figure 5:**
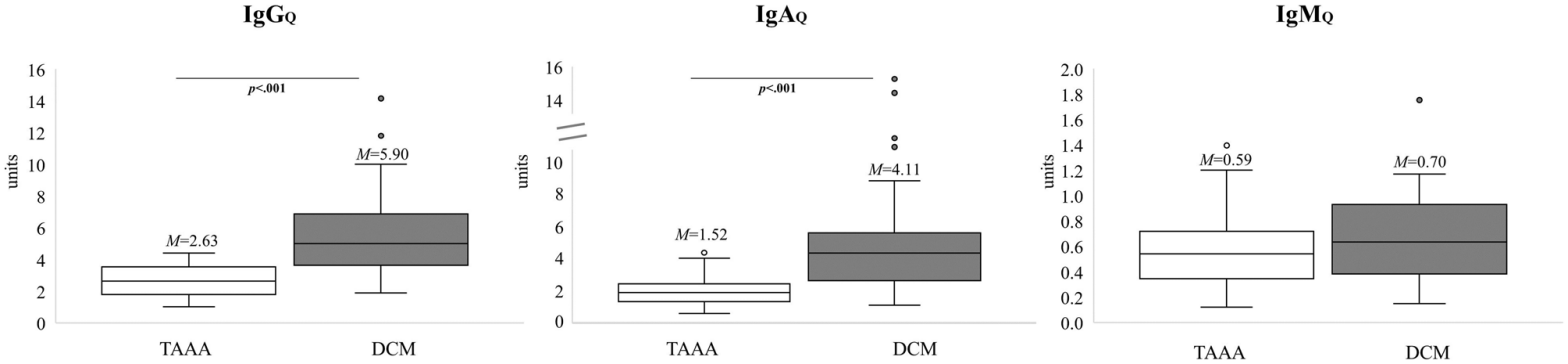
Extent of blood spinal cord barrier (BSCB) disruption preoperatively. The quotients for IgA (IgA_Q_), IgG (IgG_Q_) and IgM (IgM_Q_) are shown and compared between the control (n_IgA,IgG_ = 42, n_IgM_ = 28) and DCM groups (n_IgA,IgG_ = 43, n_IgM_ = 32). Please note the significant differences for IgA_Q_ and IgG_Q_ indicating BSCB disruption. M = mean. p = p value. TAAA = thoracic abdominal aortic aneurysm. DCM = degenerative cervical myelopathy.

The results of Independent Samples t-Tests revealed a statistically significant difference regarding the assessment of almost all CSF/serum quotients: as compared to the control group, patients showed increases in Albumin_Q_ (*p* < 0.001, Fig. 4), IgA_Q_ (*p* < 0.001, Fig. 5), and IgG_Q_ (*p* < 0.001, Fig. 5). The group difference in IgM_Q_ did not reach significance (*p* = 0.255, Fig. 5).

We next investigated a possible difference in quotients between clinically severely affected (mJOA severe = 0–11) and less severely affected patients (mJOA mild = 15–17 and mJOA moderate = 12–14). However, we could not find any significant differences for Albumin_Q_ (*p* = 0.545), IgA_Q_ (*p* = 0.975) and IgG_Q_ (*p* = 0.688).

#### Comparison of CSF/serum quotients in DCM patients with preoperative and postoperative assessment and TAAA patients

**Figure 6:**
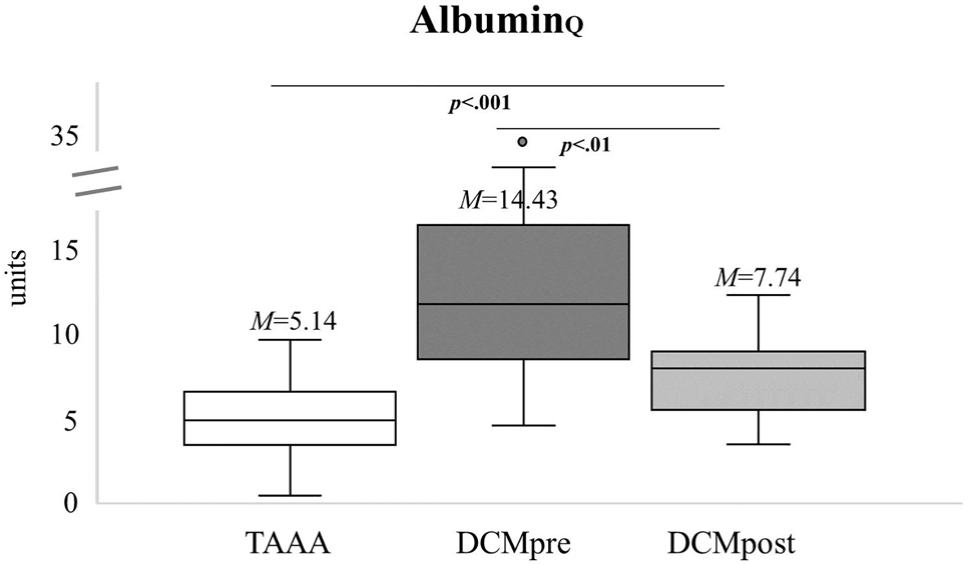
Extent of blood spinal cord barrier (BSCB) recovery postoperatively. The Albumin_Q_ of the control group (n = 43) and the DCM patients with pre- and postoperative values (n = 15) are shown. Please note the significant differences pre- and postoperatively indicating BSCB recovery. M = mean. p = p value. TAAA = thoracic abdominal aortic aneurysm. DCM = degenerative cervical myelopathy. Pre = preoperative. Post = postoperative. Albumin_Q_ = Quotient of albumin.

After surgical decompression, Albumin_Q_ was significantly decreased postoperatively compared with the mean preoperative value (*p* < 0.01 Fig. 6).

**Figure 7:**
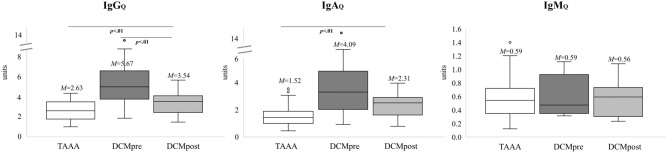
Extent of blood spinal cord barrier (BSCB) recovery postoperatively. The quotients of the control group (n_IgA,IgG_ = 42, n_IgM_ = 28) and the DCM patients with pre- and postoperative values (n_IgA,IgG_ = 15, n_IgM_ = 9) are shown. Please note the significant differences pre- and postoperatively for IgG_Q_ indicating BSCB recovery. M = mean. p = p value. TAAA = thoracic abdominal aortic aneurysm. DCM = degenerative cervical myelopathy. Pre = preoperative. Post = postoperative. IgG_Q_ = Quotient of IgG. IgA_Q_ = Quotient of IgA. IgM_Q_ = Quotient of IgM.

The same was evident for IgG_Q_ (*p* < 0.01), while IgA_*Q*_ (*p* = 0.053) and IgM_*Q*_ (*p* = 0.683) did not change significantly after surgery as shown in Fig. 7. Furthermore, the postoperative quotients remain significantly increased as compared to the TAAA control group regarding Albumin_Q_ (*p* =  < 0.001), IgG_Q_ (*p* < 0.01) and IgA_Q_ (*p* < 0.01).

### (3) Correlation between the extent of BSCB recovery and clinical improvement

**Figure 8:**
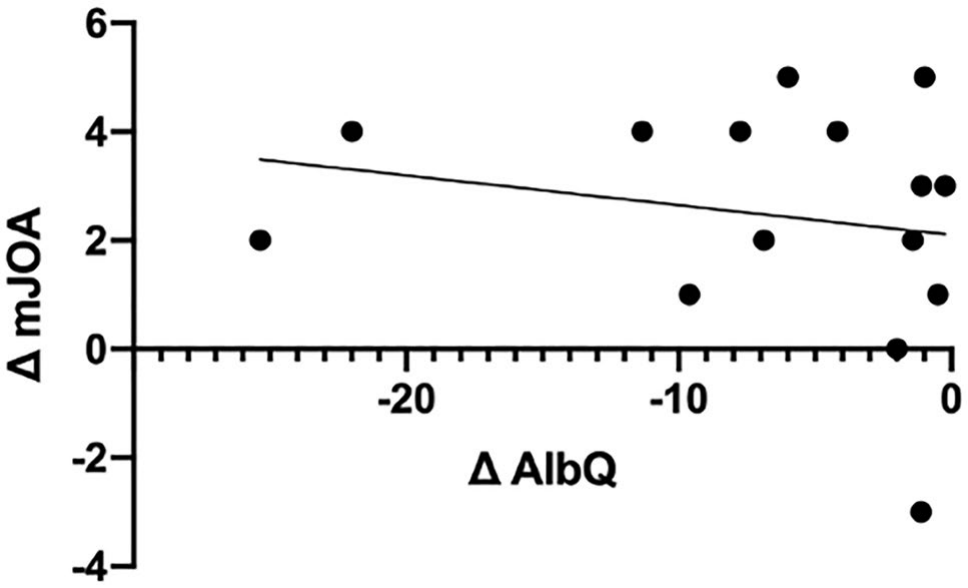
Simple linear regression model. Please note the trend towards negative linear relationship with r = − 0.2 between Δ mJOA and Δ AlbuminQ. Δ mJOA = changes in modified Japanese Orthopedic Association from postoperative to preoperative. Δ AlbQ = changes in Quotient of Albumin from postoperative to preoperative.

Table 2 shows the postoperative cases with their respective mJOA scores and quotients pre- and postoperatively. Plotting the postoperative changes in mJOA score and AlbuminQ for each patient, a trend towards a negative linear relationship with a correlation coefficient *r* = − 0.20 was found, indicating that the greater the recovery of the BSCB, the better the clinical recovery (Fig. 8).

**Table 2:** Pre-/postoperative clinical condition and quotients of DCM patients. Please note the parallel recovery of the clinical condition and BSCB disruption in the majority of cases. BSCB = Blood spinal cord barrier. NO = number. mJOA = modified Japanese Orthopedic Association. Alb_Q_ = Quotient of albumin. IgA_Q_ = Quotient of IgA. IgG_Q_ = Quotient of IgG. IgM_Q_ = Quotient of IgM.

Patient NO	Preoperatively					Postoperatively					Recovery clinic	Recovery BSCB
	mJOA	Alb_Q_	IgA_Q_	IgG_Q_	IgM_Q_	mJOA	Alb_Q_	IgA_Q_	IgG_Q_	IgM_Q_	ΔmJOA	ΔAlb_Q_
1	9	8.57	2.12	3.98	0.31	12	8.35	2.80	3.56	0.23	3	− 0.22
2	10	11.83	3.30	6.32	0.38	13	10.73	3.95	5.73	0.78	3	− 1.10
3	15	4.62	0.92	1.89	0.31	12	3.51	0.79	1.49	0.25	− 3	− 1.11
4	15	12.29	3.09	4.80	0.49	16	11.79	2.50	5.36	0.65	1	− 0.50
5	11	6.55	2.02	2.98	0.43	13	5.14	1.47	2.20	0.35	2	− 1.41
6	12	14.90	4.83	6.66	0.47	14	8.03	2.88	4.15	0.40	2	− 6.87
7	11	16.53	5.71	7.39	1.11	12	6.92	2.08	3.49	0.59	1	− 9.61
8	12	10.82	3.60	4.47	Not specif	16	6.64	1.89	2.63	0.74	4	− 4.18
9	13	34.36	18.48	28.35	Not specif	17	12.37	2.99	5.09	0.48	4	− 21.99
10	9	34.36	3.79	6.12	Not specif	11	9.01	2.90	3.82	not specif	2	− 25.35
11	9	13.29	3.75	5.45	0.93	13	5.55	2.04	3.11	1.08	4	− 7.74
12	12	11.69	3.20	5.04	0.50	17	5.70	1.62	2.46	not specif	5	− 5.99
13	12	7.28	1.96	3.07	Not specif	12	5.29	1.08	2.30	not specif	0	− 1.99
14	11	9.40	1.38	3.81	Not specif	16	8.42	3.05	3.74	0.94	5	− 0.98
15	9	19.95	6.43	8.86	0.91	13	8.60	2.65	3.92	0.68	4	− 11.35

## Discussion

While a BSCB disruption is well-known in acute SCI, its impact in chronic spinal cord disorders is rarely investigated. However, recently published studies provided first evidence that this pathomechanism may also be a key mechanism in DCM patients^12,23–25^. With an increased number of participants in this study, we confirmed our previous results of a preoperatively existing BSCB disruption according to the Reiber criteria published recently^12^. Using the Queckenstedt maneuver, we ruled out the possibility of a CSF stop in order to avoid false positive increased quotients. A significant correlation between the extend of preoperative symptoms (as indicated by mild, moderate and severe mJOA scores) and the extend of BSCB disruption could not be found. This is possibly due to the low number of patients in every severity classification, especially concerning patients with mild symptoms. At the molecular level, a BSCB breakdown can lead to a disruption of tight junction connectivity and basal lamina integrity, attributing to increased permeability^24^. The increased permeability in turn enables a secondary cascade of pathomechanisms that contributes to local spinal cord damage^3,26–29^. Preventing the development of this cascade or alleviating its severity could be an important therapeutic goal in DCM patients, as it is known that the BSCB remains chronically disrupted in untreated DCM^30^. Therefore, we also investigated the impact of surgical decompression on BSCB disruption.

The analysis of the quotient findings after surgery has not yet been described. In our study, we were able to detect a relevant BSCB recovery in 15 patients three months after surgical decompression of the spinal cord. In some cases, a BSCB disruption even restored completely according to the Reiber criteria. Our data showed a significant group difference between pre- and postoperative DCM patients for Albumin_Q_ and IgG_Q_, which tended to reach scores similar to those of the control group (Figs. 6 and 7). Although the effect can already be seen in this small sample size, it has yet to be confirmed with more patients. There is already first evidence to support BSCB recovery and axonal regeneration after surgical decompression in animal experiments^31,32^. To our knowledge, this is the first study to examine pre- and postoperative course of CSF findings in humans. Outcomes after surgical decompression for DCM have been prospectively investigated by AOSpine North America and AOSpine CSM International, indicating a sustained long-term improvement in neurological function^33,34^. The exact molecular mechanisms for this clinical recovery remain incompletely understood and require further intensive translational research. It is conceivable that not only the pathomechanisms but also the recovery processes are very similar to those in SCI. In this respect, there is already a better evidence base for SCI^26,27,35–40^. Astrocytes have the ability to reduce inflammation, cellular degeneration and restabilize the BSCB by forming a glial scar^39,40^. In addition to astrocytes, the glial scar is composed of multiple cell types such as oligodendrocyte progenitor cells, fibroblasts, macrophages and microglia interacting with each other^37,38^. This cell compound is capable of both, hindering and promoting vascular and axonal regeneration leading to BSCB reformation^37,41^. It has already been suggested in a rodent model that there is an association between restoration of BSCB and locomotor recovery after SCI due to limiting the influx of neurotoxins to the spinal cord (e.g. plasma proteins, immune cells, nitric oxide synthase)^27^.

Interestingly, we found such an association between recovery of the BSCB and improvement in the mJOA score in our postoperative DCM patients. Despite the low number of patients, our data indicate a decrease in Albumin_Q_ being accompanied with an improvement of the mJOA score. (However, it has to be reported that in one patient, the mJOA score remained the same postoperatively, but the Albumin_Q_ decreased. Another patient had a lower mJOA score postoperatively, but also a lower Albumin_Q_.) This association needs to be verified and tested for significance in further studies with a larger patient cohort. If this relationship can be confirmed, the measurement of BSCB disruption by Albumin_Q_ and Ig_Q_ could be a promising biomarker for clinical recovery. Therefore, intensive research is required to understand the chronic lesion in DCM patients, which is characterized by ongoing mechanical microtrauma and persisting BSCB disruption. We believe that the regeneration process of the BSCB is one of the crucial factors for the postoperative recovery of DCM patients and thus monitoring of this BSCB recovery could become of clinical importance.

## Limitations

This is a prospective single-center study with a relatively large DCM cohort, but it remains difficult to perform specific subgroup analyses. In particular, the correlation between the different clinical severity grades (as measured by mJOA subgroups) and the extent of BSCB disruption may require a multicenter study with a larger DCM patient cohort. Our definition of a BSCB disruption is based on the Reiber criteria for this study. An analysis of other indirect signs of BSCB disruption, such as spinal cord swelling on MRT, was not performed. Although the mJOA is a tool for describing neurological status in DCM patients in an objectifiable manner, it remains coarse and subjective. Postoperative clinical and laboratory data were collected from 15 patients three months after surgery. Later examination time points, e.g. after one year, would be of high interest, as would confirmation of these results with a larger sample size. However, this is the only study assessing postoperative CSF findings in association with BSCB in DCM patients.

## Conclusion

Our data confirm the preoperative existence of a BSCB disruption in DCM patients. This BSCB disruption has the potential to recover after surgical decompression, which is detectable using Reiber diagnostics. The possible association between the extent of BSCB recovery and clinical improvement needs further investigations. We hypothesize that monitoring of BSCB disruption may be a promising diagnostic and potential therapeutic biomarker in DCM patients, which could lead to more individualized DCM treatments in the future.

## Data Availability

The datasets used and analysed during the current study are available from the corresponding author on reasonable request.
